# Host tolerance and resistance to parasitic nest flies differs between two wild bird species

**DOI:** 10.1002/ece3.5682

**Published:** 2019-10-14

**Authors:** Kirstine M. Grab, Brian J. Hiller, John H. Hurlbert, McKenzie E. Ingram, Alexandra B. Parker, Darya Y. Pokutnaya, Sarah A. Knutie

**Affiliations:** ^1^ Department of Ecology, Evolution, and Behavior University of Minnesota Twin Cities St. Paul MN USA; ^2^ Biology Department Bemidji State University Bemidji MN USA; ^3^ Christmas Forest Bemidji MN USA; ^4^ Department of Biology University of Minnesota Morris Morris MN USA; ^5^ Department of Ecology and Evolutionary Biology University of Connecticut Storrs CT USA

**Keywords:** ecoimmunology, host defense, immune response, resistance, tolerance

## Abstract

1. Hosts have developed and evolved defense strategies to limit parasite damage. Hosts can reduce the damage that parasites cause by decreasing parasite fitness (resistance) or without affecting parasite fitness (tolerance). Because a parasite species can infect multiple host species, determining the effect of the parasite on these hosts and identifying host defense strategies can have important implications for multi‐host–parasite dynamics.
2. Over 2 years, we experimentally manipulated parasitic flies (*Protocalliphora sialia*) in the nests of tree swallows (*Tachycineta bicolor*) and eastern bluebirds (*Sialia sialis*). We then determined the effects of the parasites on the survival of nestlings and compared defense strategies between host species. We compared resistance between host species by quantifying parasite densities (number of parasites per gram of host) and measured nestling antibody levels as a mechanism of resistance. We quantified tolerance by determining the relationship between parasite density and nestling survival and blood loss by measuring hemoglobin levels (as a proxy of blood recovery) and nestling provisioning rates (as a proxy of parental compensation for resources lost to the parasite) as potential mechanisms of tolerance.
3. For bluebirds, parasite density was twice as high as for swallows. Both host species were tolerant to the effects of *P. sialia* on nestling survival at their respective parasite loads but neither species were tolerant to the blood loss to the parasite. However, swallows were more resistant to *P. sialia* compared to bluebirds, which was likely related to the higher antibody‐mediated immune response in swallow nestlings. Neither blood recovery nor parental compensation were mechanisms of tolerance.
4. Overall, these results suggest that bluebirds and swallows are both tolerant of their respective parasite loads but swallows are more resistant to the parasites. These results demonstrate that different host species have evolved similar and different defenses against the same species of parasite.

## Graphical abstract

Host defenses to parasitic nest flies differ between tree swallows and eastern bluebirds. Both host species are tolerant to their respective parasite loads but swallows are more resistance to the parasites, which is mediated by the parasite‐binding antibody response in nestlings. Photos: Jeremy Cohen.

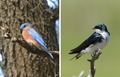

## 1 INTRODUCTION

Parasites can cause a decrease in host fitness, but hosts have developed and evolved defense mechanisms to reduce parasite damage (Clayton, Koop, Harbison, Moyer, & Bush, 2010; Lehmann, 1993; Owen, Nelson, & Clayton, 2010). Hosts can reduce parasite damage by decreasing parasite fitness (resistance) or reduce parasite damage without affecting parasite fitness (tolerance) (Medzhitov, Schneider, & Soares, 2012; Miller, White, & Boots, 2006; Råberg, Sim, & Read, 2007; Read, Graham, & Råberg, 2008; Sorci, 2013). Resistance mechanisms, such as mounting an immune response, can kill the parasite and therefore reduce the costs associated with parasite exposure, such as blood loss (Owen et al., 2010). Tolerance mechanisms, such as resource compensation or tissue repair, do not kill the parasite but instead allow the host to deal with greater parasite pressure (Christe, Richner, & Oppliger, 1996; Knutie et al., 2016; Medzhitov et al., 2012; Morrison & Johnson, 2002; Tripet & Richner, 1997). However, host defenses mechanisms can be costly, and therefore, hosts have to balance these investments with other important processes including reproduction, migration, and foraging to maximize their fitness (Graham et al., 2010, 2011; Lochmiller & Deerenberg, 2000; Sheldon & Verhulst, 1996; Van Der Most et al., 2011).

Defense strategies can differ among host species because hosts have different ecology, morphology, physiology, and behavior. Although host species can be infested with the same generalist parasite species, the effect of the parasite on host species can differ significantly (Christe, Giorgi, Vogel, & Arlettaz, 2003; Mlynarek, Knee, & Forbes, 2014; Mugabo, Decencière, Perret, Meylan, & Galliard, 2014; Rohr, Raffel, & Hall, 2010). These different outcomes among hosts and their parasites are likely related to the effectiveness of host defenses. For example, previous studies have reported that different host species can mount different immune responses, which likely affects resistance to the parasite (Lee, Martin, Hasselquist, Ricklefs, & Wikelski, 2006; Millet, Bennett, Lee, Hau, & Klasing, 2007; Palacios & Martin, 2006; Spottiswoode, 2007). Additionally, host species body size can affect their tolerance to parasitism. Despite similar parasite densities (number of parasites per gram of host), nestling birds of larger‐bodied host species are less affected by parasitic nest flies than small‐bodied hosts, suggesting that larger hosts are better defended and more tolerant of parasites than smaller hosts (Heimpel, Hillstrom, Freund, Knutie, & Clayton, 2017; Knutie et al., 2016; McNew & Clayton, 2018). Smaller‐bodied hosts have higher surface area to volume ratios and higher metabolic rates and therefore require more energy per gram of body mass than larger‐bodied hosts (Schmidt‐Nielson, 1984). These traits could increase the cost of the infection if the hosts are not able to find enough food resources to generate energy to allocate toward tolerance mechanisms, such as repairing damaged tissues or recovering lost resources, such as red blood cells. Although several studies have shown correlations between host fitness and parasite load (Careau, Thomas, & Humphries, 2010; Christe et al., 1996; Dudaniec, Kleindorfer, & Fessl, 2006), few field experiments directly compare host defenses between species against the same native parasite.

One potential model system to study how host defense mechanisms differ between species in response to the same parasite is the box‐nesting bird‐parasite system of eastern bluebirds (*Sialia sialis*) and tree swallows (*Tachycineta bicolor*) and their parasitic nest flies *Protocalliphora sialia* (DeSimone, Clotfelter, Black, & Knutie, 2018; Hannam, 2006; Roby, Brink, & Wittmann, 1992). While adult flies are nonparasitic, the larvae live in the nest and feed nonsubcutaneously on the blood of nestlings (Boyd, 1951). Several studies report no detectable lethal effects of *P. sialia* on nestling survival of tree swallows and eastern bluebirds, while others report sublethal effects of the parasite such as lower hemoglobin levels, lower body mass, and delayed fledging in parasitized nestlings compared to nonparasitized nestlings (Table 1). Despite similar varying effects of parasitism on these two host species, parasite abundance differs between them. On average, tree swallows have 36.5 ± 6.5 parasites per nest and eastern bluebirds have 81.1 ± 11.5 parasites per nest (Table 1). However, mass of the host and clutch size can affect parasite load (Dudaniec & Kleindorfer, 2009; Dudaniec et al., 2006) and eastern bluebirds have greater body mass than tree swallows while tree swallows generally have larger clutch sizes than bluebirds (Pinkowski, 1977b; Winkler et al., 2011). To control for clutch size and body mass differences between host species, parasite density (number of parasites per gram of host) can be calculated from previous studies (Table 1). We multiplied the average clutch size for each population by the average hatch mass of swallows (2.4 g) and bluebirds (3.8 g), which resulted in a total mass for the nest; average hatch mass was calculated from our Minnesota field site since these data are not available for most of the studies listed in the table. The average number of parasites published in the study was then divided by total mass of the nestlings. The average parasite density in bluebirds is still higher than swallows (Table 1; bluebirds: 4.36 ± 0.85 parasites per gram of nestling, swallows: 2.50 ± 0.49 parasites per gram of nestling). Based on these results, *P. sialia* either prefers bluebirds over swallows or each host species has evolved different defenses against the parasite.

**Table 1:** Relationship between *Protocalliphora* sp. and fledging success in eastern bluebirds and tree swallows across the United States and Canada between 1927 and 2016

Host sp.	Parasite sp.	Location	Year	Study type	Effect	Mean abundance	Mean density	Cite No.
Eastern bluebird	*Protocalliphora sialia*	Pennsylvania USA	1996–97	E	0	40.1 ± 8.8 (23)	2.65	1
	*P. sialia*	New York USA	1987–88	E	0	116.0 ± 17.2 (21)	1.20	2
	*Protocalliphora* spp.	Massachusetts USA	1927	C	−	74.4 ± NA (12)	5.34	3
	*Protocalliphora* spp.	Michigan USA	1970–74	C	−	91.4 ± 6.3 (71)	6.10	4
	*Protocalliphora* spp.	Quebec Canada	1989–90	C	0	103.8 ± 16.8 (18)	6.50	5
	*P. sialia*	New York USA	1986–88	C	0	60.8 ± NA (325)	4.36	6
Grand mean						81.1 ± 11.5 (6)	4.36 ± 0.85 (6)	
Tree swallow	*Protocalliphora* spp.	British Columbia Canada	2003	E	0	50.1 ± 8.6 (33)	3.54	7
	*P. sialia*	Massachusetts USA	2014–16	E	0	19.6 ± 2.4 (91)	1.79	8
	*P. sialia*	New York USA	1987–88	E	0	60.0 ± 10.9 (19)	1.00	2
	*Protocalliphora* spp.	Alberta Canada	2007	E	0	21.6 ± 3.8 (11)	1.54	9
	*Protocalliphora* spp.	Quebec Canada	2008–09	C	0	23.7 ± 3.7 (207)	2.12	10
	*Protocalliphora* spp.	Alberta Canada	2004	C	0	44.1 ± 5.9 (17)	3.72	11
	*Protocalliphora* spp.	Massachusetts USA	1927	C	−	55.0 ± NA (3)	4.07	3
	*Protocalliphora* spp.	Quebec Canada	1989–90	C	0	49.6 ± 8.4 (43)	4.40	5
	*P. sialia*	Nova Scotia Canada	1999	C	0	4.6 ± NA (48)	0.33	12
Grand mean						36.5 ± 6.5 (9)	2.50 ± 0.49 (9)	

Note

The types of studies were either experiment (E) or correlational (C) and found no relationship (0) or a negative relationship (−) between *Protocalliphora* spp. and fledging success. Parasite abundance is shown as the mean ± *SE* with number of nests in parentheses. Mean parasite density (number of parasites per gram of nestling) was calculated by dividing the mean parasite abundance by the average mass of nestlings in the nests from the study.

Citations: (1) Hannam (2006), (2) Roby et al. (1992), (3) Johnson (1929), (4) Pinkowski (1977a), (5) Smar (1996), (6) Wittmann and Beason (1992), (7) Dawson, Hillen, and Whitworth (2005), (8) DeSimone et al. (2018), (9) Stephenson, Hannon, and Proctor (2009) (10) Daoust, Savage, Whitworth, Bélisle, and Brodeur (2012) (11) Gentes et al. (2007) (12) Thomas and Shutler (2001).

The first goal of the study was to compare the effects of *P. sialia* on growth and survival of eastern bluebird and tree swallow nestlings in the same geographic location. Specifically, we experimentally manipulated *P. sialia* and then quantified growth metrics and fledging success of nestlings. Based on prior studies, we predicted that *P. sialia* would not significantly affect nestling growth and survival of bluebirds and swallows and therefore both host species would be effectively defended against the parasite (DeSimone et al., 2018; Gentes, Whitworth, Waldner, & Fenton, 2007; Hannam, 2006; Harriman, Dawson, Clark, Fairhurst, & Bortolotti, 2014; Roby et al., 1992; Shutler, Mullie, & Clark, 2004; Thomas & Shutler, 2001). We then tested whether bluebirds and swallows had effective defenses against *P. sialia*. Previous studies found that eastern bluebirds have higher parasite densities compared to tree swallows (Table 1), and larger‐bodied bird species, such as bluebirds, may be able to tolerate parasites more than smaller‐bodied bird species (Heimpel et al., 2017; McNew & Clayton, 2018). Because swallows have lower parasite densities than bluebirds, we predicted that swallows would be resistant to *P. sialia* compared to bluebirds.

For a potential mechanism of resistance, we quantified IgY antibody levels as a proxy of the immune response and then determined whether parasite abundance was related negatively to antibody levels (Owen et al., 2010). After the host is bitten, a series of immune pathways are activated by the host to induce the inflammatory response, leading to the production of IgY antibodies, which can bind to larval parasitic nest flies (DeSimone et al., 2018; Koop, Owen, Knutie, Aguilar, & Clayton, 2013; Owen et al., 2010). These immune molecules can negatively affect ectoparasites by causing edema (tissue swelling), which prevents the parasites from feeding from the capillaries, and damage to the parasite's tissue (e.g., via the release of proteolytic molecules from granulocytes). If swallows are more resistant to *P. sialia* than bluebirds, then we predicted that nestling tree swallows would mount a higher immune response compared to bluebirds and the immune response will be negatively correlated with parasite abundance (DeSimone et al., 2018). For mechanisms of tolerance, we quantified hemoglobin levels as a potential proxy for oxygenated red blood cells recovery (i.e., tissue repair), and parental provisioning rates to determine whether parents of parasitized nestlings were compensating for energy lost to the parasite. We predicted that parasitized bluebird nestlings would have similar hemoglobin levels to nonparasitized nestlings if they are able to recover oxygenated red blood cells as an effective tolerance mechanism. Additionally, if parasitized bluebird nestlings could recover red blood cells, we predict that hemoglobin levels would be similar across varying parasite densities. Alternatively, if parents increase feeding rates when nestlings are parasitized, the nestlings might be better able to tolerate the parasites, which would also be reflected through higher blood glucose levels in parasitized nestlings (Knutie et al., 2016).

## 2 METHODS

### 2.1 Study system

Nest boxes were monitored in Northern Minnesota near the University of Minnesota Itasca Biological Station (47°13′33″N, −95°11′42″W) from May to July in 2016–2017. Tree swallows and eastern bluebirds are abundant at the site and nest readily in artificial cavities. *Protocalliphora sialia* is the only parasitic nest fly that infests swallow and bluebird nests at this site. Tree swallows build open, cup‐shaped nests, which are made of grass and feathers, in secondary cavities (Winkler et al., 2011). The clutch size of tree swallows ranges from one to nine eggs, which are incubated for about 13–14 days, and nestlings spend an average of 20 days in the nest. Swallows feed their nestlings by placing food items in the nestling's open mouth rather than by regurgitating food and the division of labor between parents for feeding varies across their range.

Eastern bluebirds also build open‐cup nests, which are made of grasses and/or pine needles, in secondary cavities (Gowaty & Plissner, 2015). The clutch size of eastern bluebirds ranges from three to seven eggs, which are incubated for about 13–14 days, and nestlings spend 16–22 days in the nest (Gowaty & Plissner, 2015; Pinkowski, 1975). As with swallows, bluebirds feed their nestlings by placing food items in the nestling's open mouth. For bluebirds, as with swallows, both parents will feed the nestlings despite division of labor varying with geographic location.

### 2.2 Experimental manipulation of parasites

Boxes were checked once a week for nesting activity. Once eggs appeared, nests were checked every other day until nestlings hatched. At hatching, the nestlings and top liner of the nest cup (i.e., just enough material to provide a barrier between the insecticide and nestlings) were removed in order to treat the nest with either water (parasitized treatment) to allow for natural parasitism or a 1% permethrin solution to remove all parasites (nonparasitized treatment) (DeSimone et al., 2018; Knutie et al., 2016). The treatment for each species was initially determined by a coin flip, and the following nests were assigned by alternating treatment for each nest. In 2016, 12 nonparasitized and 11 parasitized swallow nests and six nonparasitized and seven parasitized bluebird nests were followed. In 2017, 13 nonparasitized and 16 parasitized swallow nests and nine nonparasitized and 11 parasitized bluebird nests were followed.

### 2.3 Nestling growth and survival

Since swallow and bluebird eggs hatch asynchronously, we also determined the age of each nestling (0–2 days old) at this time by weighing them (0.1 g) with an Ohaus CS200‐100 portable compact scale balance. When nestlings were ten days old, they were weighed (g) again and tarsus length (mm), bill length (mm), and first primary feather length (mm) were measured using Avinet plastic dial calipers. They were also banded with a numbered USFWS metal band (Master's banding permit #23623). A small blood sample (<30 µl) was taken from the brachial vein of the nestlings. When nestlings were approximately 13 days old, the boxes were checked every other day from a distance (to avoid premature fledging) to determine the fledging success and the age at which the nestlings fledged or died (>10 day old nestlings are not typically removed from the nest by the parents after they die, S.A.K. personal obs.).

### 2.4 Nestling hemoglobin and glucose

Whole blood hemoglobin was measured using a HemoCue^®^ HB +201 portable analyzer, and glucose was measured using a HemoCue^®^ Glucose 201 portable analyzer. The rest of the blood was placed on ice for up to 3 hr until it was centrifuged for 3 min at 12,000 *g* at Itasca Biological Station. Plasma and red blood cells were then stored separately in a −20°C freezer.

### 2.5 Nestling immune response

Enzyme‐linked immunosorbent assays (ELISA) were used to detect the presence of *P. sialia*‐binding antibodies (IgY) in swallow and bluebird nestling plasma, with the protocol from DeSimone et al. (2018). Ninety‐six well plates were coated with 100 µl/well of *P. sialia* protein extract (capture antigen) and diluted in carbonate coating buffer (0.05 M, pH 9.6). Plates were incubated overnight at 4°C, then washed and coated with 200 µl/well of bovine serum albumin (BSA) blocking buffer and incubated for 30 min at room temperature on an orbital table. Between each of the following steps, plates were washed three times with a Tris‐buffered saline wash solution, loaded as described, and incubated for 1 hr on an orbital table at room temperature. Plasma was 1:100 diluted with sample buffer, which was made up of BSA blocking buffer and Tween 20. Wells were loaded with 100 µl/well of individual diluted host plasma in triplicate. Plates were then loaded with 100 µl/well of Goat‐αBird‐IgG‐Heavy and Light Chain HRP (diluted 1:50,000; A140‐110P; Bethyl Laboratories). Finally, plates were loaded with 100 µl/well of peroxidase substrate (tetramethylbenzidine, TMB: Bethyl Laboratories) and incubated for exactly 20 min. The reaction was halted using 100 µl/well of stop solution (Bethyl Laboratories). Optical density (OD) was measured with a spectrophotometer (PowerWave HT; 450 nm filter; BioTek). A higher OD value was indicative of a higher IgY concentration.

On each plate, a positive control of pooled plasma from naturally parasitized nestlings was used in triplicate to correct for interplate variation (24.06%). We corrected for interplate variation by first dividing the mean OD value for the positive controls for each plate by the highest OD value among all plates then by multiplying the mean for each sample by this correction factor. In addition, each plate contained a nonspecific binding (NSB) sample in which capture antigen and detection antibody were added, but plasma was excluded. Finally, each plate included a blank sample in which only the detection antibody was added, but plasma and capture antigen were excluded. Nonspecific binding absorbance values were subtracted from the mean OD value of each sample to account background binding of the detection antibody to the capture antigen.

### 2.6 Parental behavior

In 2016, the amount of time that parents spent in the box and the frequency that they fed their offspring was quantified between 0,558 and 1,335. If more than one observation occurred in a day, the order of the nests was determined by a random number generator and/or a coin toss. Behavior was quantified when nestlings were 5 and 10 days old.

Nests were checked when the observer (K.M.G.) arrived at the nest box to make sure that it was still occupied. Once the nests were checked, there was a 15‐min waiting period after checking the box before beginning the observation period to reduce the impact of the disturbance; the observer was at least 30 m from the nest box to reduce disturbance (Tripet & Richner, 1997). The observation periods lasted between 30–60 min (mean ± *SE* = 57.27 ± 1.17 min). During the observation, we determined whether they held food in their bill when possible. The amount of time spent in the box was quantified from when the adult entered the box to when they left the box. The proportion of time spent in the box was calculated by the total time adults spent in the box divided by the total observation time in seconds. A feeding event was counted when an adult either entered the box or its head was inside the box (DeSimone et al., 2018). The frequency of feeding events was calculated by taking the total number of feeding events in an observation and dividing it by the number of minutes for the total observation period.

### 2.7 Quantifying parasites

Once nestlings died or fledged, nests were collected and stored in plastic bags. Nests were dissected and all larvae, pupae, and pupal cases were counted to determine total parasite abundance for each nest. Eclosed flies were collected and identified as *P. sialia*.

### 2.8 Statistical analyses

A negative binomial and binomial general linear model (GLM) was used to analyze the effect of parasite treatment and host species on parasite load (abundance and density) and fledging success, respectively. For each host, general linear mixed models (GLMMs) were used to analyze the effect of parasite treatment on nestling growth measurements, immune response, and blood glucose and hemoglobin levels, with nest as a random effect. We initially used year as a covariate for all models but it was excluded from all models because it did not account for a significant amount of variation. We performed log_10_ transformations to normalize the data distribution for 1st primary length, bill length, mass, hemoglobin, and glucose. Since we had two days of behavioral observations (when nestlings were different ages) in 2016, GLMMs were used to determine the effect of treatment and age on parental behavior, with nest as a random effect, for each species. For the tolerance analysis, determined the reaction norm between parasite load and host health (Simms, 2000); specifically, we used GLMs to determine the effect of parasite density and host species on fledging success and mean hemoglobin levels. Analyses were conducted in RStudio (2016, version 1.0.136), and all figures were made in Prism (2017, version 7). Analyses were conducted using GLM and GLMM functions with the lme4 package and MASS package (Bates, Maechler, Bolker, & Walker, 2015; Venables & Ripley, 2002). Probability values were calculated using log‐likelihood ratio tests using the ANOVA function in the car package (Fox & Weisberg, 2011).

## 3 RESULTS

### 3.1 Effect of parasite treatment on parasite load

**Figure 1:**
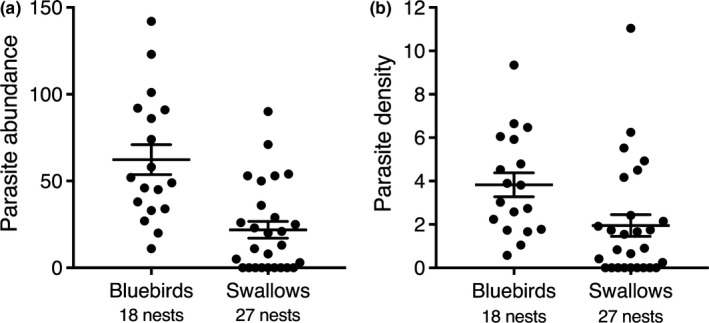
Mean ± *SE* parasite abundance (a) and density (b) of both control and experimental nests of eastern bluebirds and tree swallows across two breeding seasons

Parasite treatment reduced parasite abundance and density (which controls for host mass) in the nests of bluebirds and swallows (abundance: *χ* ^2^ = 184.55, *df* = 1, *p* < .0001; density *χ* ^2^ = 102.58, *df* = 1, *p* < .0001) (Figure 1a,b). Both swallow and bluebird nests that were treated with permethrin (nonparasitized nests) had no parasites. Swallow nests had lower parasite abundance than bluebird nests (*χ* ^2^ = 7.63, *df* = 1, *p* = .006); parasitized swallow nests had a mean ± *SE* of 21.89 ± 4.84 parasites, whereas parasitized bluebird nests had 62.33 ± 8.61 parasites (Figure 1a). Likewise, swallow nests had lower parasite density than bluebird nests (*χ* ^2^ = 5.60, *df* = 1, *p* = .02); parasite density in swallow nests was 1.95 ± 0.50 parasites per gram of mass compared to 3.83 ± 0.55 parasites per gram of mass in bluebird nests (Figure 1b). In the control treatment, the prevalence of parasites (nests that had at least one parasite) was 18/18 (100.00%) for bluebird nests and 18/27 (66.67%) for swallow nests.

### 3.2 Nestling growth and fledging success

**Figure 2:**
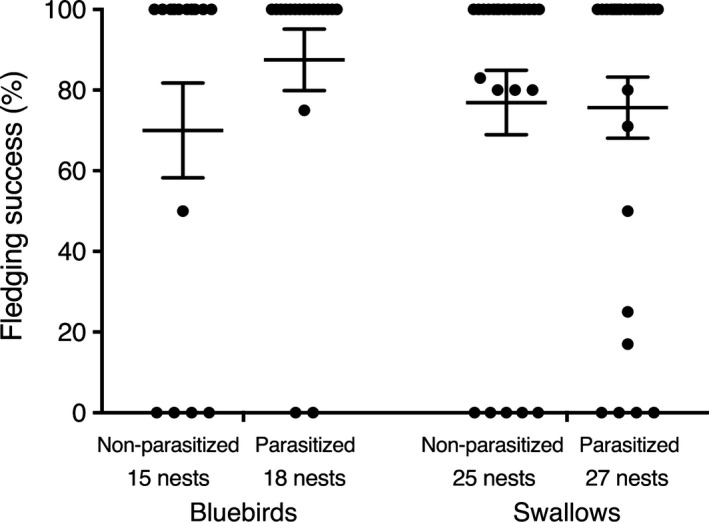
Effect of parasitism on mean ± *SE* fledging success of eastern bluebirds and tree swallows across two breeding seasons. Numbers are the number of nests per treatment and host species

For bluebirds, parasite treatment did not significantly affect bill length (*χ* ^2^ = 0.43, *df* = 1, *p* = .51), tarsus length (*χ* ^2^ = 0.51, *df* = 1, *p* = .48), 1st primary length (*χ* ^2^ = 0.01, *df* = 1, *p* = .93), mass (*χ* ^2^ = 0.03, *df* = 1, *p* = .87), or fledging success (*χ* ^2^ = 1.57, *df* = 1, *p* = .21) (Table 2, Figure 2). Similarly, for swallows, treatment did not significantly affect bill length (*χ* ^2^ = 1.26, *df* = 1, *p* = .26), tarsus length (*χ* ^2^ = 0.28, *df* = 1, *p* = .60), 1st primary length (*χ* ^2^ = 0.18, *df* = 1, *p* = .67), mass (*χ* ^2^ = 0.32, *df* = 1, *p* = .57), or fledgling success (*χ* ^2^ = 0.04, *df* = 1, *p* = .84) (Table 2, Figure 2). Overall, fledging success did not differ significantly between host species (*χ* ^2^ = 0.01, *df* = 1, *p* = .93) nor was fledging success affected by parasite density between host species (*χ* ^2^ = 0.01, *df* = 1, *p* = .91).

**Table 2:** Effect of parasite treatment on host measurements and fledging success

Measurement	Eastern bluebirds		Tree swallows	
	Parasitized	Nonparasitized	Parasitized	Nonparasitized
Bill length (mm)	5.07 ± 0.14 (18)	4.95 ± 0.12 (11)	4.43 ± 0.20 (26)	4.48 ± 0.08 (22)
Tarsus length (mm)	18.12 ± 0.32 (18)	17.82 ± 0.28 (11)	10.64 ± 0.45 (26)	11.00 ± 0.13 (22)
1st primary length (mm)	15.26 ± 1.31 (18)	13.99 ± 1.21 (11)	12.61 ± 0.97 (26)	13.12 ± 1.11 (22)
Mass (g)	25.08 ± 0.91 (18)	23.98 ± 1.37 (11)	19.34 ± 0.91 (26)	19.81 ± 0.59 (22)
Hemoglobin (g/dl)	8.91 ± 0.64 (17)	11.23 ± 0.41 (10)	10.52 ± 0.52 (24)	12.06 ± 0.29 (22)
Blood glucose levels (mg/dl)	304.44 ± 22.23 (17)	294.25 ± 13.60 (10)	276.35 ± 14.37 (23)	229.66 ± 8.52 (22)
Nestlings fledged per nest	4.11 ± 0.39 (18)	4.18 ± 0.35 (11)	4.23 ± 0.44 (26)	4.23 ± 0.35 (22)

Note

Numbers are in mean ± *SE* and numbers in parentheses are the number of nests.

### 3.3 Hemoglobin and glucose levels

**Figure 3:**
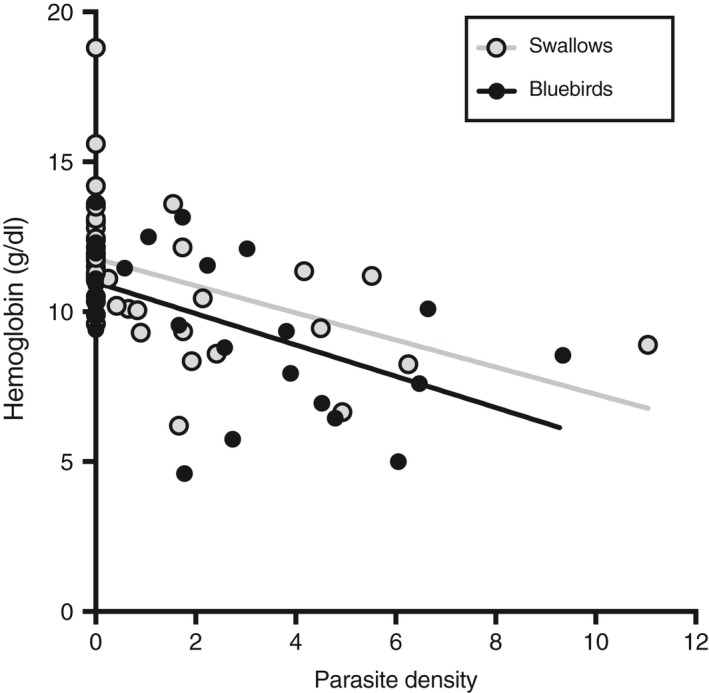
Relationship between parasite density and hemoglobin levels in eastern bluebirds and tree swallows from parasitized and nonparasitized nests

Parasitized nestlings had lower hemoglobin levels compared to nonparasitized nestlings for both bluebirds (*χ* ^2^ = 6.71, *df* = 1, *p* < .01) and swallows (*χ* ^2^ = 9.13, *df* = 1, *p* < .01) (Table 2). Parasitized swallow nestlings had higher blood glucose levels compared to nonparasitized nestlings (Table 2; *χ* ^2^ = 7.27, *df* = 1, *p* < .01) (Table 2). In contrast, glucose levels in bluebirds did not differ significantly between treatments (*χ* ^2^ = 0.00, *df* = 1, *p* = .95) (Table 2). Neither species were tolerant to parasitism with regard to blood loss; parasite density was negatively related to hemoglobin levels across species (*χ* ^2^ = 32.10, *df* = 1, *p* < .0001) but species (*χ* ^2^ = 2.61, *df* = 1, *p* = .11) and the interaction between parasite density and species (*χ* ^2^ = 0.33, *df* = 1, *p* = .56) did not affect hemoglobin levels (Figure 3).

### 3.4 Immune response

**Figure 4:**
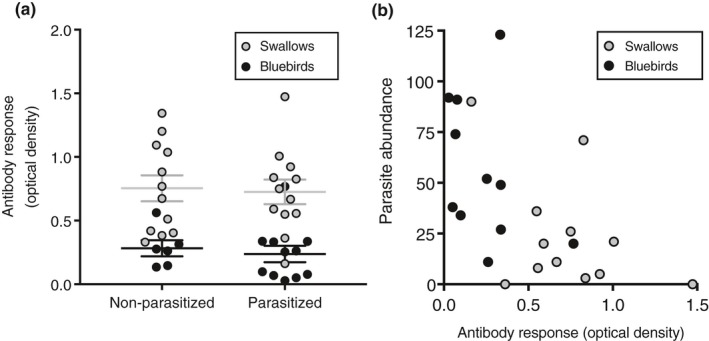
*Protocalliphora sialia*‐binding antibody response in bluebird and swallow nestlings from parasitized and nonparasitized nests in 2017. (a) Mean ± *SE* antibody response in eastern bluebirds and tree swallows for both treatments; swallows have a higher antibody response than bluebirds. (b) The relationship between parasite density and *P. sialia‐*binding antibody response in eastern bluebirds and tree swallows within the parasitized treatment. Within the parasitized nests, the antibody response is negatively related to parasite abundance in swallows but not bluebirds

Parasite treatment did not affect nestling antibody levels in bluebirds (*χ* ^2^ = 0.16, *df* = 1, *p* = .69) (Figure 4a). Bluebird antibody levels did not relate to parasite abundance (*χ* ^2^ = 2.08, *df* = 1, *p* = .15) or parasite density (*χ* ^2^ = 2.14, *df* = 1, *p* = .14) (Figure 4b). Antibody levels (optical density) in parasitized bluebird nestlings were 0.24 ± 0.06 and in nonparasitized bluebird nestlings were 0.28 ± 0.06.

Parasite treatment also did not affect significantly antibody levels (*χ* ^2^ = 0.84, *df* = 1, *p* = .36) in swallows (Figure 4a). However, antibody levels were negatively related to both parasite abundance (*χ* ^2^ = 4.49, *df* = 1, *p* = .03) and parasite density (*χ* ^2^ = 4.00, *df* = 1, *p* = .05) in swallows (Figure 4b). Antibody levels in parasitized swallow nestlings were 0.73 ± 0.10 and nonparasitized swallow nestlings were 0.95 ± 0.22. Antibody levels from parasitized nestlings differed between host species (*χ* ^2^ = 16.95, *df* = 1, *p* < .0001) (Figure 4a). The average antibody responses of swallows from parasitized nests were three times greater than those in parasitized bluebird nestlings (Figure 3a).

### 3.5 Parental behavior

The frequency with which bluebird parents fed their young was not affected significantly by parasite treatment (*χ* ^2^ = 0.03, *df* = 1, *p* = .87), nestling age (*χ* ^2^ = 1.52, *df* = 1, *p* = .22), or the effect of both treatment and age (*χ* ^2^ = 0.95, *df* = 1, *p* = .33) (Table 3). The effect of parasite treatment did not significantly affect the proportion of time parents spent in the box (*χ* ^2^ = 0.50, *df* = 1, *p* = .48), but correlated positively with nestling age (*χ* ^2^ = 10.90, *df* = 1, *p* < .001); parents spent proportionally more time inside the box with younger nestlings. The interacting effects of both age and treatment significantly impacted the time parents spent in the box (*χ* ^2^ = 9.60, *df* = 1, *p* < .01); parents spent proportionately more time in the nest when the nestlings were younger than when they were older and when the nests were parasitized compared to parents of nonparasitized nests.

**Table 3:** Effect of parasite treatment on nestling provisioning and the proportion of time that the parents spent in box

Behavioral parameter	Eastern bluebird				Tree swallow			
	Parasitized		Nonparasitized		Parasitized		Nonparasitized	
	Day 5	Day 10	Day 5	Day 10	Day 5	Day 10	Day 5	Day 10
Nestling provisioning	0.27 ± 0.08 (5)	0.26 ± 0.06 (6)	0.18 ± 0.05 (4)	0.30 ± 0.07 (4)	0.41 ± 0.03 (10)	0.42 ± 0.04 (11)	0.37 ± 0.05 (7)	0.50 ± 0.08 (8)
Proportion of time in box	0.14 ± 0.06 (5)	0.11 ± 0.06 (6)	0.07 ± 0.01 (4)	0.14 ± 0.06 (4)	0.52 ± 0.09 (10)	0.25 ± 0.08 (11)	0.71 ± 0.05 (7)	0.19 ± 0.03 (8)

Note

Proportion of time spent in the box was quantified from the total time observed. Nestling provisioning was quantified as number of feeding events per minute. Numbers are in mean ± *SE* and numbers in parentheses are the number of nests.

Feeding frequency in bluebirds did not correlate significantly with parasite abundance (*χ* ^2^ = 0.26, *df* = 1, *p* = .61), nestling age (*χ* ^2^ = 0.04, *df* = 1, *p* = .84), or the effect of age and treatment (*χ* ^2^ = 1.26, *df* = 1, *p* = .26) (Table 3). The proportion of time spent in the nest box did not correlate significantly with parasite abundance (*χ* ^2^ = 0.45, *df* = 1, *p* = .50) or nestling age (*χ* ^2^ = 0.00, *df* = 1, *p* = .98), nor was there a significant effect of the interaction of parasite abundance and nestling age (*χ* ^2^ = 3.39, *df* = 1, *p* = .07). Feeding frequency was positively correlated with blood glucose levels (*χ* ^2^ = 3.45, *df* = 1, *p* = .06), that varied across treatment (*χ* ^2^ = 0.86, *df* = 1, *p* = .35), and thus there was no effect of the interaction between treatment and glucose on feeding frequency (*χ* ^2^ = 2.51, *df* = 1, *p* = .11).

Feeding frequency in swallows did not differ significantly across treatment (*χ* ^2^ = 0.03, *df* = 1, *p* = .87) or nestling age (*χ* ^2^ = 1.52, *df* = 1, *p* = .22), nor did the interaction of nestling age and treatment have an effect on feeding frequency (*χ* ^2^ = 0.95, *df* = 1, *p* = .33) (Table 3). Parasite treatment did not affect the amount of time parents spent in the box (*χ* ^2^ = 0.50, *df* = 1, *p* = .48). However, nestling age was correlated with the proportion of time spent in the box (*χ* ^2^ = 10.90, *df* = 1, *p* < .001). The proportion of time spent in the box was affected by nestling age and varied across treatments (*χ* ^2^ = 9.60, *df* = 1, *p* < .01) as parents spent more time in boxes when the nestlings were younger compared to when the nestlings were older and they also spent more time in parasitized nests compared to nonparasitized nests.

Parasite abundance in swallows did not correlate significantly with feeding frequency (*χ* ^2^ = 0.36, *df* = 1, *p* = .55) or nestling age (*χ* ^2^ = 0.03, *df* = 1, *p* = .86), nor was the interaction between them significant (*χ* ^2^ = 0.00, *df* = 1, *p* = .99) (Table 3). Parasite abundance also did not correlate significantly with the proportion of time spent in the box (*χ* ^2^ = 0.43, *df* = 1, *p* = .51) or nestling age (*χ* ^2^ = 0.49, *df* = 1, *p* = .48) but was correlated with a decreased proportion of time spent in the box as the nestlings got older (*χ* ^2^ = 4.94, *df* = 1, *p* = .03). Swallow feeding frequency was positively correlated with blood glucose levels (*χ* ^2^ = 3.79, *df* = 1, *p* = .05), and there was no significant interaction between blood glucose levels and parasite treatment (*χ* ^2^ = 1.53, *df* = 1, *p* = .22). However, there was no significant interaction between treatment and blood glucose levels on feeding frequency (*χ* ^2^ = 0.90, *df* = 1, *p* = .34).

## 4 DISCUSSION

We examined the effects of *P. sialia* on two different species of avian hosts across two breeding seasons. *Protocalliphora sialia* did not affect the survival to fledging of either host species; however, bluebirds sustained twice as many parasites as swallows, which is consistent with the results of past studies (Table 1). Tree swallow nestlings produced an antibody response to *P. sialia*, which likely reduced parasite load (Figure 4). In contrast, bluebird nestlings did not produce a robust immune response to *P. sialia*. Both host species were tolerant to *P. sialia* at their respective parasite loads with respect to survival to fledging since increasing parasite abundances did not result in a decrease in host fitness. However, we could not determine the mechanism of tolerance. Parasitized nestlings of both species had lower hemoglobin levels than nonparasitized nestlings and were not tolerant to the blood lost to the parasite (Figure 3). Furthermore, parents from parasitized nests were not provisioning their nestlings more than parents from nonparasitized nests nor did we see differences in blood glucose levels between treatments in relation to provisioning rates (Table 3), suggesting that the rate of nestling provisioning does not increase energy compensation to the parasite. Overall, these results suggest that bluebirds are less resistant to *P. sialia* compared to swallows but both species are tolerant at their respective parasite loads.

Parasitized swallows and bluebirds did not effectively recover hemoglobin to nonparasitized levels, as found in other studies (Knutie et al., 2016; Morrison & Johnson, 2002; Råberg et al., 2007) (Table 3, Figure 3). Measuring micronuclei in red blood cells in the future would provide a proxy of whether any blood was recovered by the host (Schoenle et al., 2019). Additionally, the swallows and bluebirds might be able to quickly and effectively repair damaged epithelial tissue caused by the ectoparasite, subsequently reducing the potential for secondary infections or leaking of blood (Allen & Sutherland, 2014; Medzhitov et al., 2012; Uhazy & Arendt, 1986). This potential tolerance mechanism could be addressed in future studies by quantifying skin damage caused by the parasite and tracking the rate of repair.

While we did not see differences in feeding frequency between treatments in swallows, we found higher blood glucose levels in parasitized swallows compared to their nonparasitized counterpart. In bluebirds, there were no differences in feeding frequency or blood glucose levels between treatments. Swallows, on the other hand, were not increasing their feeding frequency to parasitized nestlings, but the nestlings still had elevated blood glucose levels. Outside of resource provisioning, blood glucose levels and subsequent glucocorticoids can also be a sign of stress. Studies have found evidence connecting parasite load to glucocorticoid and stress levels (Haond, Nolan, Ruane, Rotllant, & Wendelaar Bonga, 2003; Raouf, Smith, Brown, Wingfield, & Brown, 2006). Glucocorticoids can also regulate immune function as certain concentrations can either enhance or inhibit certain immune functions, such as immunity or inflammation (Cain & Cidlowski, 2017). Specifically, the increased blood glucose levels we detected in swallows could be a function of stress and/or another sign of an immune response. Further investigation is needed into how blood glucose levels within this system are influenced by parasites and how stress levels impact host defense mechanisms.

Parasite density was lower in swallows than bluebirds, suggesting that swallows are more resistant to the parasites than bluebirds. Parasitized swallow nestlings produced an antibody response, which was negatively related to parasite load, suggesting that swallow nestlings were able to resist the parasite to a certain load. This antibody response was likely triggered by *P. sialia* feeding on the nestlings. After the host is bitten, tissue damage and the introduction of antigens from the parasite stimulate the release of inflammatory cytokines, which triggers the migration of innate immune cells to migrate to the damaged tissue (Owen et al., 2010). These cells then degrade the antigen with the help of the major histocompatibility complex, which activates the helper T lymphocytes and the production of antigen‐specific antibodies, such as IgY antibodies. Through repeated exposure, these antibodies can quickly migrate to the wound, bind, and degrade the antigens. This immune cascade can negatively affect ectoparasites by causing edema (tissue swelling), which prevents the parasites from feeding from the capillaries, and damage to the parasite's tissue (e.g., via the release of proteolytic molecules from granulocytes).

Interestingly, however, the immune response of nestling swallows did not differ between treatments suggesting that nonparasitized birds also produced an immune response. The antibody isotype (IgY) that we quantified binds to *P. sialia* but is not specific to *P. sialia*. One possible explanation for why nonparasitized nestlings are producing an immune response is that there are other parasites in the system, such as endo‐ or intracellular parasites, that may not be affected by the experimental manipulation (Pedersen & Fenton, 2015; Shutler et al., 2004). Several studies have shown that a reduction of the target parasite resulted in an increase in a nontarget parasite species (Knowles et al., 2013; Pedersen & Antonovics, 2013). Swallows at other locations are infected with other parasites, such as the blood parasite *Trypanosoma* spp. (Shutler et al., 2004), which might not be as affected by the insecticidal treatment (Sholdt, Schreck, Mwangelwa, Nondo, & Siachinji, 1989). Such parasites might induce a nonspecific IgY immune response in the host, which could result in a significant antibody response. Future studies are needed to characterize other parasites in the birds and determine the specificity of the IgY response.

One remaining question is why are swallows more immunologically resistant to the parasite compared to bluebirds? Fassbinder‐Orth et al. (2016) found that antibodies can bind with different affinity to detection antibodies, and therefore, it is possible that bluebird antibodies have a low affinity to the detection antibody used in our study. However, a recent study found that bluebird females and nestlings supplemented with mealworms do produce an IgY response (i.e., have similarly high OD values) (Knutie, 2019), and therefore, detection antibody binding is likely similar across species. Alternatively, studies have found that larger‐bodied host species can withstand the effects of parasitism of *Philornis* spp. better than smaller‐bodied host species (Knutie et al., 2016; McNew & Clayton, 2018). Smaller‐bodied hosts have a higher surface area to volume ratio and higher metabolic requirements than larger‐bodied hosts (Furness & Speakman, 2008; Schmidt‐Nielson, 1984). The cost of infection might be higher for smaller‐bodied hosts because of their higher metabolism and energy requirements per gram of body mass (Brace et al., 2017; Furness & Speakman, 2008; Schmidt‐Nielson, 1984). Studies have documented metabolic rates increasing in the presence of parasites and parasitism incurring energy costs on the host as the parasite burden increases (Careau et al., 2010; Connors & Nickol, 1991; Møller, Lope, Moreno, González, & Pérez, 1994). Thus, even if hosts have similar parasite densities, smaller hosts might reach their maximum energy level faster than larger‐bodied host species potentially increasing the cost of infection (Brace et al., 2017; Furness & Speakman, 2008; Schmidt‐Nielson, 1984). Additionally, larger‐bodied hosts have more surface area and cells for parasites to occupy allowing for a higher parasite capacity and are able to sacrifice more resources to the parasite potentially reducing the cost of infection (Brace et al., 2017; Downs, Schoenle, Han, Harrison, & Martin, 2019). Bluebirds are larger than swallows and have higher parasite densities, supporting the idea that larger hosts can deal with more parasites. Therefore, smaller‐bodied hosts, such as swallows, might only be able to tolerate a certain parasite load before investing in resistance. Additionally, previous studies have also found that the nutritional value of nestlings' food affected their host defense strategy (De Neve et al., 2007; Knutie, 2019; O'Brien & Dawson, 2008). For example, food supplementation increased antibody production and parasite resistance in eastern bluebirds, which was possibly mediated by the gut microbiota of the host (Knutie, 2019). Therefore, it is possible that swallows are feeding their nestlings food with higher nutritional value or have gut microbiota that better primes the development of the immune system, compared to bluebirds, but these ideas need to be tested in the future.

The differences in parasite density between the two species could also be influenced by the preference of the parasite. Parasites use visual, olfactory, and chemical cues, such as CO_2_ or pheromones, to find their hosts (Chaisson & Hallem, 2012; Gold & Dahlsten, 1989; Horn, Mierzejewski, & Luong, 2018; Lehane, 2005). Because swallows and bluebirds are different sizes, they differ in their metabolic rate and thus the rate of CO_2_ release, which might make one host more attractive than the other host. Despite swallows requiring more energy per gram, bluebirds have a higher resting metabolic rate because they are larger and therefore release more CO_2_ making them more attractive to *P. sialia* than swallows (Chaisson & Hallem, 2012; Furness & Speakman, 2008; Lehane, 2005). Additionally, adult plumage color may also play a role in *P. sialia* preference for bluebirds (Lehane, 2005). Bluebirds have a different UV chroma coloration than swallows which might serve an attractant to *P. sialia* to the nest box (Bitton & Dawson, 2008; Liu, Siefferman, & Hill, 2007). Other mechanisms by which *P. sialia* might prefer bluebirds over swallows are nest characteristics. For example, certain plants contain volatile compounds that may deter parasites (Dubiec, Góźdź, & Mazgajski, 2013). Both species incorporate grasses and pine needles into their nests (Gowaty & Plissner, 2015; Winkler et al., 2011) but the effect of the specific plant composition has not been explored.

Overall, our study suggests that nestling survival of bluebirds and swallows is relatively unaffected by the *P. sialia* because the hosts can effectively defend themselves against the parasite. For example, both bluebirds and swallows can tolerate their respective loads. However, swallows sustain fewer parasites per gram of body mass compared to bluebirds, which is likely because swallows resist the parasite with an immunological response. This resistance in swallows could be because they are unable to tolerate similar parasite densities as bluebirds due to their body size because smaller‐bodied hosts probably suffer a higher cost of parasitism (Cardon, Loot, Grenouillet, & Blanchet, 2011; McNew & Clayton, 2018). Other studies have also found that bluebirds had higher parasite densities than swallows but the effect of the parasite on the hosts differs based on location and year (Table 1). Our results suggest that different host species can defend themselves similarly and differently to the same parasite, which is likely due to variation in host ecology and life history. Future studies are needed to determine if there is long‐term interannual variation in these host–parasite relationships and whether environmental factors, such as precipitation, could affect them (Musgrave, Bartlow, & Fair, 2019).

## CONFLICT OF INTEREST

The authors declare no competing interests.

## AUTHOR CONTRIBUTIONS

All authors collected data. K.M.G. and S.A.K. analyzed data and wrote the manuscript. All authors contributed to editing the manuscript.

## Data Availability

Data will be available at FigShare upon acceptance (https://doi.org/10.6084/m9.figshare.9785246).
